# Using host and bacterial genetic approaches to define virulence strategies and protective immunity during *Mycobacterium tuberculosis* infection

**DOI:** 10.1128/msphere.00517-24

**Published:** 2025-04-22

**Authors:** Andrew J. Olive

**Affiliations:** 1 Department of Microbiology, Genetics, and Immunology, College of Osteopathic Medicine, Michigan State University 43977 , East Lansing, Michigan, USA; University of Kentucky College of Medicine, Lexington, Kentucky, USA

**Keywords:** *Mycobacterium*, genetics, host-pathogen interactions, immune evasion

## Abstract

Infections with *Mycobacterium tuberculosis* (Mtb) resulted in over one million deaths in 2024, the highest number for any infectious disease. With no vaccines that protect against pulmonary tuberculosis (TB) and the challenges associated with antibiotic therapy, there is a critical need to better understand host-Mtb interactions to help curb this major public health problem. Mtb is arguably the most successful human pathogen, and it survives in diverse environments, resulting in heterogeneous disease outcomes in patients. Five years ago, in my commentary in mSphere, I discussed how Mtb virulence strategies that sense, adapt, and evade killing in the host can be uncovered using genetic approaches. Here, I will come full circle to highlight genetic approaches that recently uncovered new mechanisms regulating protective host responses and Mtb survival tactics. The goal is to highlight a genetic framework to probe a range of unexplored Mtb phenotypes, increase our understanding of host-Mtb interactions, and identify new therapeutic targets that may help prevent TB.

## INTRODUCTION

**Fig 1:**
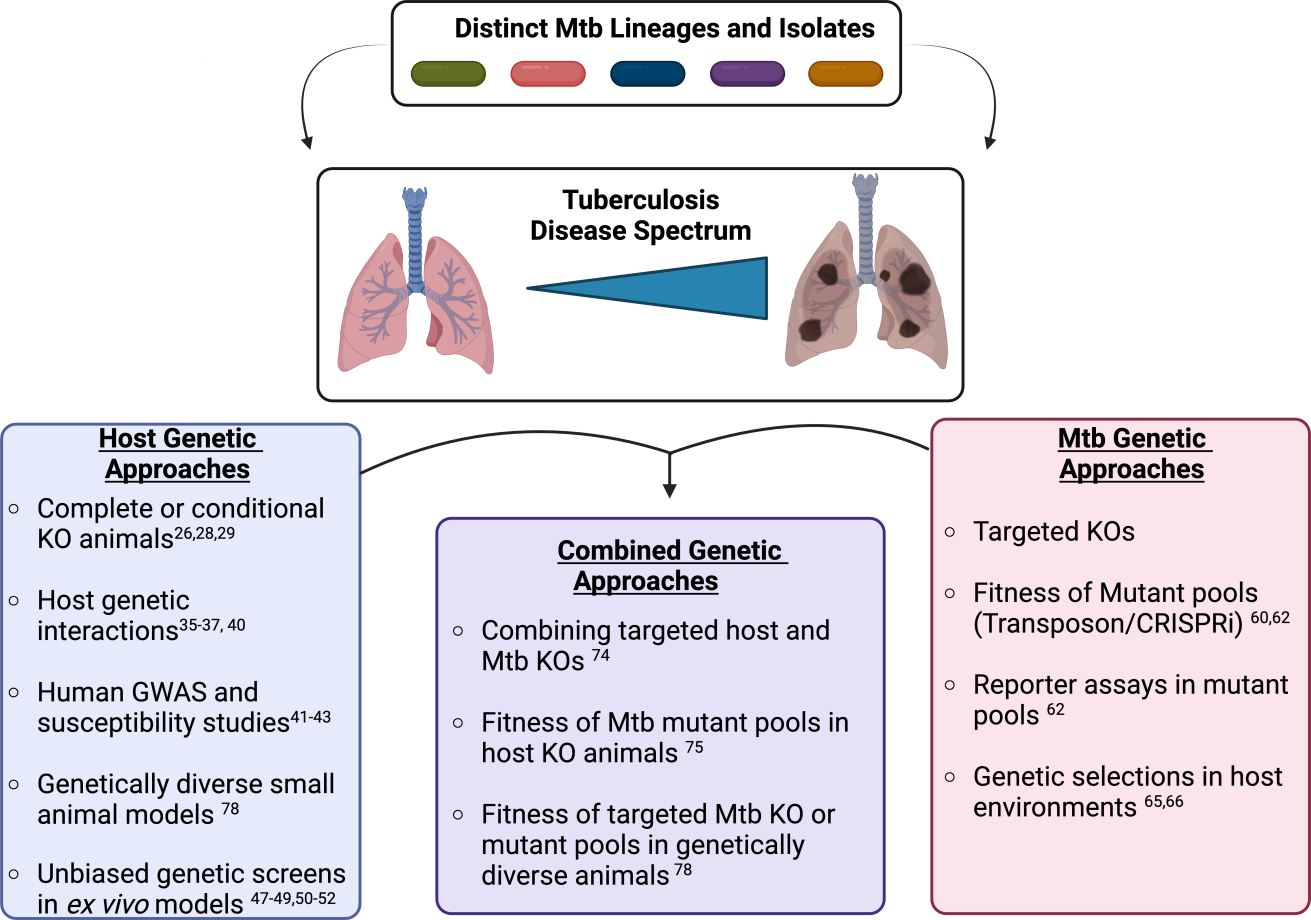
Dissecting the host and Mtb genetic diversity to understand the heterogeneity of tuberculosis disease. The combination of genetically distinct Mtb clinical isolates with the genetically variable human population results in a wide spectrum of tuberculosis disease states. These states range from asymptomatic latent disease to active disease with caseating lesions and severe immunopathology. Genetic approaches provide an opportunity to define both bacterial and host pathways that contribute to disease or protection during Mtb infection. Using either host or bacterial genetic approaches offers a detailed understanding of pathways contributing to important host-Mtb interactions. Combining host and bacterial genetic approaches can help to directly link host and bacterial networks with protection or disease. Figure made using Biorender.

The mammalian immune response is a carefully orchestrated set of interactions that allow the host to detect, respond to, and eradicate pathogens without damaging the surrounding host tissues (1). However, intracellular bacterial pathogens, such as *Mycobacterium tuberculosis* (Mtb), use an array of tactics to manipulate and withstand normal host responses, which poses major challenges to eradication and prevention (2, 3). Disruption of these complex host-pathogen interactions therapeutically requires a mechanistic understanding of the host response and immune evasion tactics. Genetic approaches provide an opportunity to define pathways in both the host and pathogen that contribute to protection or disease. Five years ago, in my mSphere of influence commentary, I discussed a particularly impactful genetic study by Rubin and colleagues, which used a “genetics-squared” approach by combining host and Mtb genetic approaches to uncover bacterial tryptophan metabolism as a potential therapeutic target during infection (4–6). I also explained how this study helped shape the framework for my own, recently started, independent research group. Since my commentary, the frontiers of genetic approaches continue to expand, further increasing our understanding of critical host-Mtb interactions. In this minireview, I will come full circle to explore these recent studies while introducing genetic approaches that may help to deconvolve the genetic diversity of both the host and pathogen to identify new targets for therapies that prevent TB disease in patients at the highest risk (Fig. 1). Importantly, this is not meant to be a comprehensive review of Mtb pathogenesis research but rather to highlight a genetic framework that can be applied to better understand Mtb-host interactions and their role in disease progression.

## MTB INFECTION AND PATHOGENESIS

Mtb is an obligate human pathogen that continues to be the leading cause of infectious diseasemediated death worldwide, with over 1 million lives taken each year (3). Following inhalation, Mtb infects airway resident alveolar macrophages (AMs) that provide a unique replicative niche for Mtb, allowing it to evade clearance, prevent robust inflammation, and delay T cell responses (7, 8). Mtb adapts to the host environment using parallel two-component regulatory systems, including PhoPR and DosRST, that sense changes in pH and oxidative stress and alter Mtb metabolic networks (9, 10). These environmental sensors control the expression of key virulence determinants such as the ESX-1 type VII secretion system that is required for intracellular Mtb survival (11). After a week or more of replication in AMs, Mtb transitions to infect recruited myeloid-derived macrophages and dendritic cells, which are more restrictive to Mtb growth and associated with host metabolic shifts toward glycolysis (12, 13). Infection of these cells also results in the robust activation of adaptive immune responses that are characterized by CD4+ and CD8+ T cells that produce IFNγ and IL-17, both of which contribute to control Mtb infection (14–16). While the adaptive immune response slows Mtb growth, it is generally insufficient to drive sterilization, resulting in the formation of granulomas that contain infected cells that are walled off from the remaining healthy lung tissue. These granulomas are complex structures with immune cells, epithelial cells, and blood vessels that all contribute to altering the environment encountered by Mtb (17–19). Changes to the environment inside the granuloma include the emergence of foamy macrophages that provide lipid reservoirs for Mtb, low oxygen (hypoxia) that drives non-replicating persistence, and necrosis of host cells that drives Mtb replication in anticipation of spread to others through cough (20–22).

Challenges to tuberculosis (TB) treatment include the heterogeneity of disease where 5%–10% of infected individuals will progress to active disease (23). In addition to inter-host variation, intra-host variation occurs where a single host may contain both actively replicating Mtb and non-replicating persistent Mtb due to the distinct trajectories of individual granulomas (24). This heterogeneity highlights the exquisite adaptability of Mtb, which also contributes to antibiotic resistance that extends treatment times for patients undergoing drug therapy (25). In the absence of vaccines that effectively prevent pulmonary disease in adults, there is an important need to understand the underlying host- and pathogen-dependent mechanisms that drive distinct disease states. In the last 5 years, several studies addressed key gaps in our understanding of Mtb-host interactions using cutting-edge genetic approaches. Together, these new studies highlight the power of genetics to uncover new protective mechanisms and dissect host-pathogen interactions that contribute to Mtb pathogenesis and TB disease.

## USING HOST GENETIC APPROACHES TO DEFINE IMMUNE PATHWAYS REGULATING MTB DISEASE PROGRESSION

### Using host genetic knockouts to dissect the contribution of candidate genes to TB protection or disease

The standard genetic approach to examine the contribution of host pathways in controlling TB disease is to leverage mice that are deficient in genes of interest in the entire animal or using conditional gene disruption strategies, such as Cre-Lox systems, that disrupt genes of interest only in a subset of cells. These animals are then challenged with Mtb, and changes in disease severity and bacterial growth compared to control animals are determined. The simple elegance of this approach continues to provide new discoveries into distinct facets of host-Mtb interactions, including providing insights into long-standing gaps in our knowledge related to early Mtb infection. One study by Rothchild et al. (26) examined the open question of why AMs do not immediately trigger inflammation and adaptive immune activation following Mtb infection. Using host RNA sequencing analysis of AMs early after Mtb infection, the authors identified the oxidative stress regulator NRF2 as a pathway that is robustly induced following infection. Using conditional knockout mice, they showed that *Nrf2* expression in AMs is required to restrain early inflammatory responses during Mtb infection and drive more rapid Mtb replication in AMs. This important finding highlights the unique inflammatory regulation of AMs and suggests that targeting AMs may accelerate adaptive immune responses.

Knockout approaches not only enable the characterization of known genes of interest but also allow the careful dissection of genetic loci associated with disease to identify causative genes. This approach was recently used to dissect the “susceptibility to tuberculosis locus 1” (SST1) that was identified in C3HeB/FeJ mice (also known as Kramnick mice) and backcrossed into the C57BL6 background (27). Yet, the causative genes of SST1 remained speculative. While previous studies predicted that the causative gene for TB susceptibility in this locus was SP110, standard recombination mouse genetic approaches were unable to specifically delete SP110 due to the highly repetitive nature of SP110 and surrounding genes. To overcome this shortcoming, the Vance lab used CRISPR-Cas9 approaches to generate loss-of-function alleles of SP110 and the neighboring gene SP140 (28). Surprisingly, they found that the loss of SP140 alone drove TB susceptibility due to increased type I IFN production. This finding suggests that SP140 and not SP110 is a key mediator of the SST1 locus and susceptibility to TB. Future work can now examine how SP140 negatively regulates IFN responses and controls susceptibility to Mtb infection.

One possible caveat of knockout animals is incomplete CRE-mediated recombination of conditional alleles, resulting in incomplete deletion. This was recently observed when Golovkine et al. (29) re-examined key regulators of autophagy. They found that hemizygous expression of LysM-Cre with floxed alleles of the autophagy regulators *Atg7* or *Atg16* does not result in complete recombination and thus did not uncover major roles for these genes in TB protection. However, increasing the CRE dosage drove more efficient gene knockout, resulting in increased early bacterial growth and decreased long-term survival of animals. Thus, autophagy genes beyond ATG5 play a role in regulating TB control and inflammatory disease. Over the last 5 years, similar approaches using knockout animals have been used to identify key roles for other host pathways in TB control, including Phospholipase C and NF-κB signaling, highlighting the complexity of the host response to Mtb infection and providing new insights into immune regulation (30, 31).

### Leveraging host genetic interactions to define parallel immune networks controlling TB progression

While knockout animals in single genes are helpful to identify pathways that contribute to TB protection, understanding how these pathways interact with or function in parallel to other immune pathways is critical to develop host-directed therapies with limited off-target effects. A useful approach to define the crosstalk between pathways is genetic interaction studies where the disease progression of two single genetic knockouts and the combination of both mutations in a double knockout are compared to wild-type animals. In some situations, a deleterious phenotype of one single knockout animal can be unchanged, improved, or exacerbated by a second knockout, which defines the potential role of each gene in TB protection or disease. This genetic interaction approach was recently used by the Vance lab to show that loss of SP140 leads to TB susceptibility through increased type I IFN production by generating double knockout mice deficient in both SP140 and the IFN receptor (IFNAR) (28). These double knockout animals were no longer susceptible to Mtb, suggesting that the causative factor driving TB disease in SP140deficient mice is dependent on type I IFN signaling.

Genetic interaction studies also recently helped to define the susceptibility observed in the absence of the cell-autonomous immunity regulator *Irgm1*. In humans, mutations in *Irgm* are associated with TB and other inflammatory diseases (32, 33). Previous work found that mice deficient in *Irgm1* are highly susceptible to Mtb due to defects in direct antimicrobial restriction and the adaptive immune response. However, subsequent studies found that IRGM1 may not directly target Mtbcontaining vacuoles, suggesting a continued lack of understanding of how IRGMs regulate immune control of TB disease (34). One study by Wilburn et al. (35) found that removing a second IRGM, *Irgm3*, in an *Irgm1-*deficient mouse reversed the TB susceptibility observed in a single *Irgm1* knockout animal. Further experiments also showed that loss of all three Irgms (*Irgm1*, *Irgm2*, and *Irgm3*) results in no appreciable differences in bacterial control and only shows a very late survival defect. Thus, genetic interaction approaches suggest that the balance between IRGMs is critical for Mtb defense rather than IRGMs directly controlling Mtb growth. To understand the underlying mechanisms of how dysregulation of IRGMs results in TB susceptibility, two elegant studies combined Irgm1-deficient mice with mice lacking *Ifnar* or *Irgm3* (36, 37). The results showed that loss of IRGM1 results in an IRGM3-dependent increase in type I IFN, which drives TB susceptibility. The similarity of these findings to those observed in *Sp140*^/−^ mice suggests that there are distinct paths for susceptibility to TB, but several paths may converge to drive a deleterious and excessive type I IFN response.

Genetic interaction approaches can also uncover unexpected TB susceptibilities. Previous studies showed that the loss of the NADPH phagocyte oxidase (Phox) results in decreased TB survival that is driven by increased inflammatory cytokines and neutrophil influx without any major changes in bacterial control (38, 39). To understand this disease tolerance phenotype, Phoxdeficient mice were crossed to mice deficient in a key regulator of the cytokine IL1, Caspase 1. While we predicted that mice lacking both Phox and Caspase 1 would be protected following Mtb infection, we instead observed that these mice were hyper-susceptible to TB, succumbing to disease within 4 weeks of infection (40). This suggests an unexpected yet strong exacerbating genetic interaction between Phox and Caspase 1 in mice. While the mechanisms underlying this susceptibility remain unclear, these striking results suggest a continued lack of understanding of how essential immune pathways intersect during infection. Phox and Caspase 1 are some of the most studied immune networks in the immunological literature, and yet, key aspects of their regulation and function remain unknown. Thus, genetic interaction studies should be more broadly employed to better understand the crosstalk between key immune regulatory networks during Mtb infection.

### Modeling human susceptibility alleles in cells and animals

Given the heterogeneity of TB outcomes in the human population, leveraging the genetic diversity of humans to TB susceptibility is an important genetic approach. These large-scale human projects use genome-wide association studies and single nucleotide polymorphism analysis to find alleles that are associated with the highest risk of disease. These studies have found several associations, yet most remain to be replicated in other populations (41–43). Another human genetic approach is to identify patients with susceptibilities of unknown etiology and define the mechanisms and genes underlying their susceptibility. One recent study used this approach to define the susceptibility of two adult cousins from Colombia with recurrent pulmonary TB disease (44). Surprisingly, genetic analysis found that these patients had a loss-of-function mutation that led to a complete deficiency in the protective cytokine TNF. TNF is associated with protection from a range of diseases, yet these patients, beyond their TB susceptibility, were otherwise healthy and did not develop any adverse reactions following BCG immunizations. The power of this study stems from using cells from the patients and using normal iPSCs to generate isogenic TNFdeficient human macrophages. This study raises many important questions, including what protective pathways act redundantly to TNF to protect against other pathogens and what differences between *Mycobacterium bovis* BCG and Mtb drive differential survival to hostderived ROS. More broadly, this surprising finding highlights new avenues to understand how immune pathways interact with and compensate for one another.

Beyond using *ex vivo* approaches to validate human genetic disease alleles, other approaches include introducing human disease-associated alleles into small animal models. One recent example of this approach is the study from the Watson and Patrick labs, which used a human diseaseassociated gain-of-function allele in the gene leucine-rich repeat kinase 2 (LRRK2) (45). They observed that the disease-associated G2019S allele drove mitochondrial dysfunction and gasdermin D-dependent necroptosis in macrophages following Mtb infection. This resulted in hyperinflammation and immunopathology, highlighting a key role for mitochondrial maintenance in balancing the pulmonary inflammatory response during TB. Taken together, these studies highlight the strengths of human genetic approaches in making new inroads in understanding what mediates host protection during Mtb infection.

### Using host-based forward genetic screens to uncover new regulators of inflammation and Mtb control

While using targeted genetic approaches in animal models can define the protective function of host genes of interest, this approach is limited by the need for candidate genes to study in the first place. Leveraging unbiased forward genetic screens provides an opportunity to uncover new candidate genes that may play important roles in distinct aspects of the host response to Mtb infection. Gene editing approaches using CRISPR-Cas9 tools enable the generation of genome-wide loss-of-function or gain-of-function libraries in a wide range of human or mouse cell lines (46). These libraries can then be used to identify genetic perturbations that alter phenotypes of interest. Several recent studies have used CRISPR-Cas9generated libraries to define genes regulating cellular responses that are important during Mtb infection.

### Identifying genes that modulate infection phenotypes

To date, three published studies have used CRISPR Cas9 screens to examine host pathways regulating cellular responses to Mycobacterial infection in macrophages (47–49). The first study, from the Nathan lab (47), used a genome-wide screen in RAW264.7 cells. They isolated genes that, when lost, lead to increased cellular survival 5 days following Mtb infection. Their study described an important role for type I IFN in modulating Mtb-dependent cell death responses and found that blocking IFN signaling can augment antibiotic therapy *in vivo*. Similarly, a second study by the Lu lab (48) used both knockout and knockdown libraries in human Thp1 cells and found that blocking IFN improved cell survival following infection with *M. bovis* BCG. The authors additionally discovered a key role for the aryl hydrocarbon receptor (AHR) pathways in driving cell death following *M. bovis* BCG infection. To validate their genetic findings, this study leveraged chemical inhibitors of these pathways as an orthologous approach. The drugs cerdulantinib and CH223191 that target type I IFN and AHR, respectively, result in increased Mtb control and host cell survival. Finally, in a recent study, Simwela et al. (49) used a viability-based cell sorting screen in conditionally immortalized HoxB8 bone marrow-derived macrophages (BMDMs) to identify host pathways that, when lost, lead to less cell death in part due to reduced Mtb replication. They uncovered a new role for the E3 ubiquitin ligase, glucose-induced degradation/C-terminal to LisH complex, in suppressing intracellular Mtb nutrient and oxidative stress. Together, these studies highlight that cell death mechanisms are a critical target for host-directed therapies. Yet, these pathways are very complex and require more mechanistic studies to fully understand their regulation in mice and humans. Second, while these studies primarily use cell death-/survival-based readouts, it is easy to imagine leveraging these forward genetic tools in the future to define other pathways involved in Mtb-host phenotypes, including the activation of inflammatory cytokines, induction of Mtb two-component regulators, and other important host-pathogen interaction pathways.

### Defining genes required for effective responses to protective cytokines

Mtb is uniquely adapted to thwart normally effective host responses (50). Yet, the precise mechanisms of how host regulatory pathways are manipulated are generally unclear. An important consideration in our quest to identify protective host-direct therapy targets is to better understand how protective immune pathways normally tune cytokine responses to drive bacterial control without augmenting inflammatory damage. One such cytokine is IFNγ. Loss of IFNγ results in severe susceptibility to Mtb infection in humans and animal models, yet IFNγ is also insufficient to drive Mtb eradication from infected hosts (51, 52). These data suggest that Mtb may evade a subset of IFNγ-dependent mechanisms to survive *in vivo*. However, successful treatments will not be as simple as increasing the production of IFNγ to drive protection, as data from the Barber lab (53) strongly suggests that increasing IFNγ on a per-cell basis worsens TB disease. Thus, understanding how distinct aspects of IFNγ-dependent responses are regulated may provide more targeted opportunities to increase the capacity of TB control without increasing pathologic inflammation.

To better understand IFNγ regulatory mechanisms, investigators are using CRISPR screens to define IFNγ regulatory networks in macrophages in normal conditions. One study examined genes that regulate the IFNγ-dependent expression of NOS2, finding a new role for the post-translational modification UFMylation in negatively regulating IFNγ responses (54). How the loss of UFMylation alters Mtb infection and disease remains to be fully understood, but should be tested in the future. In parallel to this study, our research group examined the shared and unique regulation of the T cell modulatory markers MHCII, CD40, and PD-L1 that are induced on the surface of macrophages following IFNγ activation (55). Among shared regulators, we found a surprising requirement for Complex I of the electron transport chain to induce robust IFNγ responses. While the mechanisms are not yet clear, it is possible that pools of NAD+ that are products of Complex I enzymatic function help macrophages respond to IFNγ. Given that Mtb encodes enzymes that degrade NAD+, this pathway could be further understood in the context of previous studies, suggesting that Mtb alters IFNγ responses (56). We also examined specific regulators of MHCII, uncovering an important role for the glycerol synthase kinase 3α and β (GSK3α/β) in responding to IFNγ (57). GSK3α/β are important regulators of inflammation and resolution, and a recent chemical inhibitor study found that targeting GSK3α/β may alter Mtb pathogenesis *ex vivo* (58). Thus, a more detailed understanding of GSK3α/β regulation and function during Mtb infection is critically important. Taken together, these approaches help underscore how new genetic approaches can be leveraged to dissect the regulation of protective pathways like IFNγ in order to improve Mtb control and overcome immune evasion strategies.

## USING BACTERIAL GENETIC APPROACHES TO DEFINE VIRULENCE STRATEGIES REQUIRED FOR MTB PERSISTENCE AND DISEASE PROGRESSION

While defining protective host immune responses is important, it is equally important to understand how Mtb senses and adapts to the ever-changing host environment. Bacterial genetic approaches provide an important strategy to define the requirement of distinct bacterial pathways that regulate Mtb survival in different growth conditions and environments. This includes understanding how distinct Mtb lineages are associated with different immune evasion mechanisms and inflammatory responses. Over the last 5 years, several research groups leveraged Mtb genetic approaches in creative ways to uncover new mechanisms of pathogenesis and immune evasion.

### Defining bacterial pathways that modulate Mtb survival strategies

Genome-wide bacterial genetic approaches like transposon sequencing (TnSeq) and CRISPRi continue to illuminate important regulatory mechanisms controlling Mtb pathogenesis (reviewed in reference 59). Recent modification to these tried-and-true bacterial genetic approaches provides new insights into Mtb stress responses, metabolism, and physiology. Previously, a key caveat in TnSeq experiments has been the inability to query the role of essential central metabolism genes during infection. In a recent study, the Tischler lab overcame this barrier by using a modified growth media with supplemented nutrients to generate a transposon library containing essential metabolic pathways (60). This library led to the finding that Mtb requires phenylalanine synthesis and purine/thiamine synthesis to survive in the lungs of animals. In addition to TnSeq experiments, genome-wide Mtb CRISPRi screens also allow genetic interrogation into the regulation of Mtb stress responses through transient CRISPR-mediated knockdown. WhiB7 is a critical transcriptional regulator induced in stress conditions and is responsible for inducing several antibiotic resistance mechanisms in Mtb (61). One recent report coupled a CRISPRi library with a fluorescent reporter of WhiB7 expression (62). Using this clever approach, the authors identified Mtb genes that, when lost, result in increased WhiB7 expression even in non-stress conditions. This genetic screen highlights the important role of amino acid levels in the stress response of Mtb. It found that WhiB7 responds to alanine levels through a direct transcriptional feedback loop involving the alanine biosynthesis enzyme AspC. Together, these unbiased genome-wide studies suggest that targeting Mtbspecific amino acid synthetic pathways may disrupt Mtb growth and persistence.

### Identifying genes that contribute to Mtb persistence in host-like conditions

While WhiB7 regulates several stress responses, Mtb coordinates other responses to adapt to life inside macrophages, including lower pH, changes in oxygen, and oxidative stress. In response to these environmental changes, Mtb adapts its transcriptome and metabolism to slow its replication, enabling persistence (63). The two-component regulator PhoPR is central to low-pH adaptation, driving growth arrest in a pH-dependent manner. Interestingly, *in vitro* studies found that Mtb only arrests growth in low pH when grown in particular carbon sources, such as glycerol, but the underlying mechanisms remained unclear (64). To address this gap, the Abramovitch group (65, 66) recently conducted a genetic selection to identify Mtb variants that do not arrest growth in low pH but rather show enhanced growth in acidic conditions when glycerol is the sole carbon source. They found a range of point mutations in the PE/PPE protein PPE51, and mechanistic follow-up studies revealed that these mutations result in increased glycerol uptake. In line with other recent reports, this suggests that PPE51 can serve as a glycerol transporter and that tuning glycerol uptake at acidic pH can drive Mtb growth arrest or replication (67). This genetically regulated growth arrest in response to low pH is important for pathogenesis, as PPE51 mutants with enhanced acidic growth are better controlled by activated macrophages. This study highlights how a well-designed genetic selection can interrogate the regulation of key growth arrest strategies employed by Mtb in the host. Future work can expand this approach to test a range of stress conditions to define how Mtb persists to cause disease.

### Using Mtb clinical isolates to define mechanisms of pathogenesis and disease

While TnSeq, CRISPRi, and genetic selections are powerful approaches to understand Mtb pathogenesis and immune evasion, these studies historically use a single laboratoryadapted Mtb strain, such as H37Rv or Erdman. However, recent data strongly suggest evolution among distinct Mtb lineages that have important effects on TB disease (68, 69). In support of this model is a recent study from the Tobin lab (70) that found an Mtb strain causing an outbreak in North Carolina with unusually high rates of extrapulmonary dissemination. Sequencing analysis of the outbreak strain identified it as a lineage 1 strain and harboring a full-length functional allele of EsxM, a secreted effector associated with the ESX-V secretion system. In contrast, they found that modern Mtb lineages 2–4 introduced a stop codon in EsxM, suggesting selective evolution of a loss-of-function mutation in this gene. Mechanistic studies found that EsxM coordinates macrophages’ migration and egress from granulomas, which likely contributes to extrapulmonary disease. These data suggest that modern Mtb lineages drive less dissemination and skeletal disease and are an important example of considering how distinct Mtb lineages and clinical isolates contribute to the heterogeneity of Mtb disease outcomes.

To characterize distinct disease trajectories and immune evasion tactics by different Mtb lineages more broadly, Carey et al. (71) developed a molecular barcoding strategy to track individual lineage strains from a pool containing 16 clinical isolates from lineages 2, 3, and 4 following infections of the mouse lungs. They found that isolates from distinct lineages had similar growth dynamics, with lineage 2 strains showing slow growth and lineage 4 strains showing rapid growth over the first 2 weeks of infection. These differences in growth dynamics may contribute to changes in Mtb disease and protection, as lineage 2 isolates were less controlled by BCG vaccination in animals. The differences between isolates were further characterized by determining transcriptional changes of each isolate grown in distinct *in vitro* stress conditions, including ROS, low pH, and nutrient starvation and characterizing the genetic requirements of distinct clinical isolates using TnSeq in mice. These studies found that differentially required genes between isolates are generally regulated by transcription factors with strain-specific variants, such as KstR, which regulates cholesterol metabolism in Mtb. Thus, the ongoing evolution of Mtb in human populations is resulting in changes to the wiring and requirement of distinct metabolic pathways associated with intracellular survival.

## COMBINING HOST AND BACTERIAL GENETIC APPROACHES TO CONNECT HOST-PATHOGEN INTERACTIONS THAT CONTRIBUTE TO TB PROTECTION OR DISEASE

While using host or bacterial genetics approaches continues to uncover new insights into Mtb pathogenesis and the host response, it is important to consider how distinct combinations of Mtb strains and host genotypes interact to cause heterogeneous disease outcomes. Over the last 5 years, several studies combined host and pathogen genetics approaches to dissect new host-Mtb interactions in more detail than ever. The strength of this “genetics-squared” approach is reviewed thoroughly by Persson and Vance (6), but the key importance is the ability to directly connect host pathways with bacterial pathways. This approach can identify immune evasion tactics, innate immune sensing networks and their ligands, as well as define key metabolic interactions that occur during Mtb infection.

### Dissecting immune evasion tactics using targeted host and Mtb genetic approaches

It is clear that Mtb evades a wide range of normally effective immune responses, given that the majority of immunocompetent hosts cannot eradicate Mtb. Understanding how these evasion tactics function can uncover new targets for therapy that drive improved bacterial control. One area of interest in therapeutic development is targeting distinct host cell death modalities that are associated with protection (i.e., apoptosis) versus pathology (i.e., necrosis) (72). It is known that the activation of the NLRP3 inflammasome during Mtb infection can drive cell death by pyroptosis and increase IL-1 production that can contribute to TB disease, yet the interactions between Mtb and this pathway remain unclear (73). A recent study from Rastogi et al. (74) found that Mtb inhibits the activation of the NLRP3 inflammasome independently of the ESX-1 secretion system. Using a candidate gene, PknF, that was identified in a previous cell death screen, the authors found that loss of PknF results in increased IL-1β production by BMDMs. However, this is dependent on caspase 1 as infection of caspase 1/11 and caspase 1 knockout macrophages with a ΔPknF strain results in no IL-1β production. Future work will be needed to determine how modulating pyroptosis by blocking PknF function alters Mtb infection kinetics and protective immunity. These experiments combining host and bacterial knockouts help to directly connect the function of PknF with NLRP3, IL-1β production, and pyroptosis. Thus, combining specific host and bacterial knockouts is an effective approach to define new immune evasion strategies employed by Mtb.

### Identifying new Mtb immune evasion tactics combining genome-wide Mtb genetic approaches with defined immune-deficient hosts

Understanding what Mtb genes contribute to evasion of particular cell-autonomous immune pathways, such as autophagy, is critical to identify new therapeutic targets. The Philips research group (75) recently defined what Mtb genes are required for survival in autophagy-sufficient cells. Using a genome-wide transposon library, wild-type, *Atg5*^−/−^, and *Atg7^−/−^* BMDMs were infected, and changes in the representation of the transposon library were defined by TnSeq analysis. Interestingly, they found an important role for phthiocerol dimycocerosate (PDIM) in enabling Mtb to resist autophagic-dependent killing. In mice, PDIM was required for Mtb to survive in myeloid-derived cells when autophagy was functional, linking the *ex vivo* screen with *in vivo* relevance. These data suggest that one mechanism by which Mtb evades autophagy is through the action of PDIM, a known virulence determinant. More broadly, the approach used in this report shows the strength of unbiased forward bacterial genetic screens to directly identify new immune evasion tactics.

### Globally identifying host-pathogen interactions by combining genome-wide bacterial genetic approaches with large-scale models of host genetic diversity

The diversity of TB progression in human patients is highly variable. Yet, modeling this diversity in animal models is challenging. Throughout this review, the majority of studies use mice with genetic manipulations in a C57BL6 background that are normally resistant to TB. Yet, studies from the C3HeB/FeJ model clearly show that introducing changes in the host genetic background can model distinct manifestations of tuberculosis disease (27). Thus, more broadly modeling genetic diversity in the host has the potential to uncover new pathways that contribute to distinct Mtb infection outcomes.

Over the last decade, Mtb researchers have used different models of genetic diversity, including the diversity outbred mouse model, the BXD model, and the collaborative cross (CC) (76–78). The CC contains over 70 strains that are a distinct mosaic of five inbred and three wild-derived mouse strains, and each strain is reproducible as recombinant inbred mice that are genome sequenced (79). In a recent large-scale study, the Sassetti and Smith labs showed that infection of CC mice with Mtb results in a broad diversity of disease outcomes (78). In some cases, TB survival was associated with bacterial levels; however, a subset of strains succumbed to disease with low bacterial load, while others harbored high bacterial loads with little disease symptoms. In this single study alone, nine high-confidence and suggestive genetic loci were associated with Mtb disease phenotypes, including spleen CFU, IL-17 production, and CXCL1 secretion. Future studies can now examine strains with distinct disease trajectories and dissect the causative genes associated with quantitative traits.

In addition to characterizing host disease phenotypes, the CC mice in this study were infected with a genomewide Mtb transposon library, enabling the global identification of bacterial genes that are required in distinct CC strains. The analysis from this experiment shows that the total number of Mtb genes that are required in mice expanded from ~200 genes in C57BL6 mice to ~750 genes total across the CC panel. In many cases, unique bacterial pathways were required only in a subset of CC mice, suggesting new host-Mtb connections to examine experimentally. An additional strength of this experimental approach is using the fitness scores of individual Mtb mutants in the transposon screen as quantitative traits to identify what is termed “host interacting with pathogen QTL” (HipQTL). Over 40 HipQTLs were identified, and they contained bacterial pathways, including the ESX1 secretion system, mycobactin, and the Mce4 operon that is associated with cholesterol uptake. This novel genetic analysis will identify immune evasion strategies by connecting key virulence determinants with particular host loci. Future work can now take candidate gene approaches to dissect individual HipQTLs and better understand host pathways that can help to effectively control Mtb pathogenesis and disease.

## LOOKING TO THE FUTURE

The recalcitrance of Mtb to antibiotic therapy and the difficulty in developing a broadly protective vaccine require a deeper understanding of Mtb pathogenesis and the protective host response. It is clear that Mtb is well adapted to evade normally protective immune mechanisms while also adapting to the rapidly changing host environment. Understanding the bacterial and host pathways at these interfaces will be critical to develop therapies that decrease treatment time and/or drive long-lasting protection. In this full circle minireview, I highlighted how genetic approaches were used to identify new host and bacterial pathways that are important in Mtb pathogenesis and disease. Future work can now apply these genetic approaches to probe more deeply into host-Mtb interactions. These future applications may include (i) developing genetic approaches in other host cell models that represent distinct lung immune cells, (ii) using other bacterial transcriptional or stress reporters to characterize the regulation of essential Mtb adaptations, and (iii) employing new innovative models of genetic diversity and gene editing. In addition, new frontiers in host and bacterial genetics are enabling household contact studies to identify factors that contribute to Mtb resistance and impact transmissibility (80, 81). Altogether, innovations and applications with both host and Mtb genetic approaches will help continue to provide new insights to make progress in the important public health fight against TB.
